# The burden of carbon monoxide poisoning in China from 1990 to 2021 and forecasts to 2050

**DOI:** 10.3389/fpubh.2025.1642416

**Published:** 2025-08-25

**Authors:** Yanwu Yu, Jinzhou Yu, Ding Yuan, Fang Yang, Hongyi Yan, Pin Jiang, Mengnan Guo, Zhigao Xu, Gaiqin Yan, Yan Zhang, Yanxia Gao

**Affiliations:** ^1^Department of Emergency, The First Affiliated Hospital of Zhengzhou University, Zhengzhou, China; ^2^School of Nursing, Li Ka Shing Faculty of Medicine, The University of Hong Kong, Hong Kong, Hong Kong SAR, China

**Keywords:** carbon monoxide poisoning, the Global Burden of Diseases, Injuries, and Risk Factors Study (GBD), incidence, prevalence, mortality, Disability Adjusted Life Years

## Abstract

**Aim:**

This study aimed to analyze the disease burden of carbon monoxide poisoning (COP) in China from 1990 to 2021 and to forecast future trends.

**Methods:**

Data were retrieved from the Global Burden of Diseases, Injuries, and Risk Factors Study (GBD) 2021. The incidence, prevalence, mortality, and Disability Adjusted Life Years (DALYs) and their corresponding Age-Standardized Rates (ASRs) were examined to assess the burden of COP in China from 1990 to 2021. The Joinpoint regression model was used to investigate the trend changes of COP burden. The Age-Period-Cohort model was employed to delineate age, period, and cohort effects on the trends in disease burden. The Bayesian Age-Period-Cohort analysis method was applied to predict the changing trends of COP disease burden in China from 2022 to 2050.

**Results:**

In 2021, there were 152,820 incidences COP in China, with an Age-Standardized Incidence Rate (ASIR) of 13.06 per 100,000 population. There were 13,289 fatalities, corresponding to an Age-Standardized Mortality Rate (ASMR) of 0.8 per 100,000 population. The total DALYs amounted to 499,528, with an Age-Standardized DALY Rate (ASDR) of 35.02 per 100,000 population. The peak burden, as measured by DALYs, was observed in the 30–34 age group. Trend analysis employing Joinpoint regression revealed an initial increase in ASIR and Age-Standardized Prevalence Rate (ASPR) until 2015, followed by a subsequent decline. Moreover, the ASMR and ASDR showed fluctuant downward trends. Predictions from the Bayesian Age-Period-Cohort model suggest that the incidence, prevalence, mortality, and DALYs of COP in China are expected to increase from 2022 to 2050, peaking in 2038, while the ASRs are projected to decline.

**Conclusion:**

From 1990 to 2021, China experienced dynamic temporal patterns in the burden of COP, characterized by an initial rise in incidence and prevalence, followed by a decline in recent years, alongside a general downward trajectory in mortality and DALYs. Additionally, projections indicate a potential resurgence in COP-related burden in the coming decades.

## 1 Introduction

Carbon monoxide (CO), commonly referred to as the “silent killer,” poses severe health risks due to its capacity to elude detection while inflicting lethal harm. Carbon monoxide poisoning (COP) disrupts oxygen supply and utilization, induces oxidative stress, and impairs cellular functions, which can lead to outcomes as severe as loss of consciousness, acute cardiac events, and even death (1, 2). Furthermore, for individuals who survive the acute stage of poisoning, there is a decreased long-term survival rate, a potential for neurological sequelae in later stages, and an increased likelihood of cardiovascular disease, respiratory disease, and other systemic diseases, imposing substantial burden on families and healthcare systems (3).

CO is produced ubiquitously during the incomplete combustion of various fuels in both household and industrial settings (4, 5). Common sources include coal, water heaters, generators, cooking appliances, gasoline engines, and fire incidents (6). This widespread prevalence makes COP a recurring and significant global public health concern. Annually, US emergency departments report approximately 50,000 acute COP cases, resulting in over 1,000 fatalities (7). The latest Global Burden of Diseases, Injuries, and Risk Factors Study (GBD) analysis results indicate that in 2021, the estimated global COP-related deaths totaled 28,900 (95% uncertainty interval (UI), 21,700–32,800), with a mortality rate of 0.366 (0.276–0.415) per 100,000 population (8). However, the mortality rate of East Asia was 0.802 per 100,000 (0.465 to 0.962), with 13,500 (7,690 to 16,600) across all ages, surpassing the global level (8). As the largest country in East Asia, the COP disease burden in China has yet to be estimated.

Over the past 30 years, China, as the world’s largest developing country, has experienced rapid development, with a continuous transition in energy application. The proportion of clean energy has significantly increased, while the proportion of coal application has significantly decreased. This energy application transformation could potentially reduce CO sources, thereby decreasing COP incidence. Consequently, this study, based on the 2021 GBD data, examines the changing pattern of the disease burden caused by COP in China from 1990 to 2021 and forecasts COP’s epidemic trend from 2022 to 2050, providing data support for COP prevention and control in China and other developing countries.

## 2 Materials and methods

### 2.1 Data source

Data for this study was derived from the GBD 2021, which evaluated the disease burden associated with 369 diseases and injuries, complications, and 87 risk factors across 204 countries and territories from 1990 to 2021 (8). The ICD-10 codes used were X47-X47.9. COP was defined as accidental poisoning from exposure to CO, excluding intentional poisoning. The epidemiological indicators selected to assess the burden of COP included incidence, prevalence, deaths, and Disability-Adjusted Life Years (DALYs). We extracted data stratified by age and sex for China from 1990 to 2021, along with their 95% UI. This study is compliant with GATHER. This manuscript was produced in accordance with the GBD Protocol.

### 2.2 Measurement indicators

The burden of COP in China was evaluated using the number of incidences, prevalence, deaths, and DALYs, as well as their corresponding Age-Standardized Rates (ASRs). These include Age-Standardized Incidence Rate (ASIR), Age-Standardized Prevalence Rate (ASPR), Age-Standardized Mortality Rate (ASMR), and Age-Standardized DALYs rate (ASDR). DALYs represent a combined measure of premature death and disability, expressed in person-years to represent the length of survival time.

### 2.3 Statistical methods

Data visualization was performed using R software version 4.3.3. Annual Percent Change (APC) and Average Annual Percent Change (AAPC) for each indicator were calculated using Joinpoint software version 5.0.2 to analyze trends in the disease burden of COP. The Age-Period-Cohort model analysis tool provided by the National Cancer Institute website was further used to explore the age, period, and cohort effects on the changing patterns of COP disease burden. Lastly, a Bayesian Age-Period-Cohort analysis method was applied to predict the changing trends of COP disease burden in China from 2022 to 2050, based on the epidemiological data of COP in China from the GBD 1990–2021 study. The future standardized population was projected using the population data from the GBD 2021 study database.

### 2.4 Joinpoint regression analysis

The Joinpoint regression model is a statistical model used to investigate the trend changes of the indicators over time. It identifies potential inflection points, assisting researchers in determining the growth rate, directionality, and location of these points within different time intervals. By fitting straight line segments and inflection points, the model can provide a depiction and prediction of the variable trends. The modeling method for Joinpoint regression is the Grid Search Method (9, 10).

### 2.5 Age-period-cohort model analysis

The Age-Period-Cohort model, predicated on the Poisson distribution, enhances traditional descriptive analysis methods by addressing the collinearity issue inherent among age, period, and cohort variables. This model facilitates the examination of these three elements as independent factors influencing disease incidence (11, 12). In this study, epidemiological data for COP were arranged in five-year intervals from 1992 to 2021, and in five-year age groups, according to the characteristics of the GBD 2021 data and Age-Period-Cohort model web analysis tool. In all Age-Period-Cohort analyzes, the middle age group, period, and birth cohort were used as references.

### 2.6 Bayesian age-period-cohort analysis

The Bayesian Age-Period-Cohort model estimates the future distribution by integrating sample information with prior information about unknown parameters, then inferring these parameters. This predictive process is implemented using the BAPC and INLA packages in R (13, 14).

## 3 Results

### 3.1 Burden of COP in China in 2021

In 2021, there were 152,820 (95% UI, 112,293–199,602) new cases of COP in China, with an ASIR of 13.06 (95% UI, 9.39–17.3) per 100,000 population. In the same period, there were 206,771 (95% UI, 169,171–247,573) prevalent cases, with an ASPR of 12.27 (95% UI, 9.99–14.66) per 100,000. The number of deaths due to COP was 13,289 (95% UI, 6,544–17,340), with an ASMR of 0.8 (95% UI, 0.39–1.03) per 100,000. The DALYs attributed to COP was 499,528 (95% UI, 255,310–642,013), with an ASDR of 35.02 (95% UI, 17.83–44.18) per 100,000 (Table 1).

**Table 1:** Incidence, prevalence, mortality, DALYs and corresponding ASRs of COP in China in 2021.

Age	Sex	Incidence		Prevalence		Deaths		DALYs	
		Number	ASIR	Number	ASPR	Number	ASMR	Number	ASDR
All ages	Both	152,820 (112293–199,602)	13.06 (9.39–17.3)	206,771 (169171–247,573)	12.27 (9.99–14.66)	13,289 (6544–17,340)	0.8 (0.39–1.03)	499,528 (255310–642,013)	35.02 (17.83–44.18)
	Female	63,509 (46109–83,716)	11.38 (8.03–15.45)	76,933 (62299–92,508)	9.17 (7.4–11.05)	4,539 (1103–6,272)	0.54 (0.14–0.74)	162,401 (43973–220,628)	23.98 (6.69–32.01)
	Male	89,312 (66151–115,934)	14.66 (10.72–19.27)	129,838 (106249–154,952)	15.35 (12.56–18.22)	8,750 (4695–12,186)	1.08 (0.58–1.48)	337,128 (184301–464,342)	45.63 (24.37–61.81)
<5	Both	5,165 (3419–7,691)	6.65 (4.4–9.9)	743 (579–928)	0.96 (0.75–1.19)	283 (118–426)	0.36 (0.15–0.55)	25,016 (10509–37,599)	32.21 (13.53–48.41)
	Female	1859 (1198–2,784)	5.16 (3.32–7.72)	224 (171–285)	0.62 (0.47–0.79)	109 (30–180)	0.3 (0.08–0.5)	9,672 (2716–15,903)	26.84 (7.54–44.13)
	Male	3,306 (2209–4,935)	7.94 (5.31–11.85)	519 (408–644)	1.25 (0.98–1.55)	173 (73–266)	0.42 (0.18–0.64)	15,344 (6504–23,488)	36.86 (15.62–56.42)
5–9	Both	12,603 (6800–20,315)	13.16 (7.1–21.21)	2,879 (2003–3,800)	3.01 (2.09–3.97)	265 (118–357)	0.28 (0.12–0.37)	22,267 (10158–29,925)	23.25 (10.61–31.25)
	Female	5,001 (2554–8,315)	11.16 (5.7–18.55)	931 (641–1,248)	2.08 (1.43–2.78)	111 (37–154)	0.25 (0.08–0.34)	9,303 (3206–12,882)	20.75 (7.15–28.74)
	Male	7,602 (4221–12,174)	14.92 (8.29–23.9)	1948 (1359–2,552)	3.82 (2.67–5.01)	154 (67–218)	0.3 (0.13–0.43)	12,964 (5847–18,225)	25.45 (11.48–35.78)
10–14	Both	16,418 (7853–28,106)	19.05 (9.11–32.61)	4,513 (3420–6,132)	5.24 (3.97–7.11)	255 (127–327)	0.3 (0.15–0.38)	20,196 (10319–25,787)	23.43 (11.97–29.92)
	Female	7,256 (3448–12,554)	18.05 (8.58–31.23)	1,570 (1177–2,143)	3.9 (2.93–5.33)	106 (33–143)	0.26 (0.08–0.36)	8,383 (2702–11,197)	20.85 (6.72–27.85)
	Male	9,162 (4426–15,586)	19.92 (9.62–33.89)	2,943 (2245–3,981)	6.4 (4.88–8.65)	149 (71–202)	0.32 (0.15–0.44)	11,813 (5869–15,864)	25.68 (12.76–34.49)
15–19	Both	18,529 (8630–32,409)	24.81 (11.56–43.4)	5,905 (4475–7,866)	7.91 (5.99–10.53)	342 (183–439)	0.46 (0.25–0.59)	25,273 (13802–32,347)	33.85 (18.48–43.32)
	Female	8,248 (3771–14,519)	23.85 (10.9–41.98)	2,111 (1573–2,849)	6.1 (4.55–8.24)	128 (34–168)	0.37 (0.1–0.49)	9,469 (2698–12,387)	27.38 (7.8–35.81)
	Male	10,281 (4863–18,197)	25.65 (12.13–45.4)	3,793 (2888–5,047)	9.46 (7.2–12.59)	214 (118–297)	0.53 (0.29–0.74)	15,804 (8899–21,830)	39.43 (22.2–54.46)
20–24	Both	20,151 (10266–34,537)	27.54 (14.03–47.2)	7,787 (5984–10,388)	10.64 (8.18–14.2)	514 (258–682)	0.7 (0.35–0.93)	35,335 (18162–46,809)	48.29 (24.82–63.97)
	Female	8,533 (4219–15,067)	24.87 (12.3–43.92)	2,815 (2118–3,805)	8.21 (6.17–11.09)	154 (33–224)	0.45 (0.1–0.65)	10,642 (2520–15,295)	31.02 (7.35–44.58)
	Male	11,618 (6035–19,469)	29.89 (15.53–50.09)	4,971 (3859–6,562)	12.79 (9.93–16.88)	360 (169–517)	0.93 (0.43–1.33)	24,693 (11821–35,326)	63.53 (30.41–90.89)
25–29	Both	19,909 (9616–35,154)	23.02 (11.12–40.65)	11,330 (8771–14,333)	13.1 (10.14–16.57)	558 (281–730)	0.65 (0.33–0.84)	35,674 (18441–46,666)	41.25 (21.32–53.96)
	Female	7,952 (3672–14,505)	19.46 (8.99–35.49)	4,074 (3113–5,239)	9.97 (7.62–12.82)	138 (32–212)	0.34 (0.08–0.52)	8,949 (2189–13,519)	21.9 (5.36–33.08)
	Male	11,957 (5920–20,707)	26.21 (12.98–45.39)	7,257 (5650–9,064)	15.91 (12.39–19.87)	420 (204–593)	0.92 (0.45–1.3)	26,725 (13298–37,587)	58.59 (29.15–82.4)
30–34	Both	18,815 (8407–32,673)	15.53 (6.94–26.97)	19,425 (15195–23,616)	16.03 (12.54–19.49)	733 (374–978)	0.6 (0.31–0.81)	43,329 (22759–57,461)	35.76 (18.79–47.43)
	Female	7,223 (3043–12,607)	12.35 (5.21–21.56)	6,947 (5398–8,597)	11.88 (9.23–14.7)	201 (40–316)	0.34 (0.07–0.54)	11,977 (2767–18,635)	20.48 (4.73–31.87)
	Male	11,592 (5281–20,226)	18.49 (8.43–32.27)	12,478 (9814–15,211)	19.91 (15.66–24.27)	532 (279–763)	0.85 (0.44–1.22)	31,352 (16702–44,789)	50.02 (26.64–71.45)
35–39	Both	9,468 (3872–17,990)	8.94 (3.65–16.98)	18,469 (14694–22,488)	17.43 (13.87–21.22)	669 (341–907)	0.63 (0.32–0.86)	36,356 (19090–49,154)	34.31 (18.02–46.39)
	Female	3,650 (1400–7,287)	7.07 (2.71–14.12)	6,614 (5183–8,079)	12.82 (10.04–15.66)	178 (36–268)	0.34 (0.07–0.52)	9,772 (2262–14,543)	18.94 (4.38–28.18)
	Male	5,819 (2438–11,014)	10.7 (4.48–20.26)	11,855 (9503–14,370)	21.81 (17.48–26.43)	491 (246–730)	0.9 (0.45–1.34)	26,584 (13717–39,352)	48.9 (25.23–72.38)
40–44	Both	4,438 (1791–8,455)	4.85 (1.96–9.24)	16,220 (13057–19,621)	17.72 (14.27–21.44)	637 (346–867)	0.7 (0.38–0.95)	31,341 (17355–42,474)	34.24 (18.96–46.4)
	Female	1,699 (666–3,238)	3.81 (1.49–7.26)	5,811 (4604–7,035)	13.03 (10.32–15.77)	173 (31–259)	0.39 (0.07–0.58)	8,598 (1870–12,763)	19.27 (4.19–28.61)
	Male	2,739 (1132–5,168)	5.84 (2.41–11.01)	10,409 (8410–12,631)	22.18 (17.92–26.92)	464 (248–666)	0.99 (0.53–1.42)	22,743 (12474–32,499)	48.47 (26.58–69.26)
45–49	Both	3,850 (1575–7,177)	3.49 (1.43–6.51)	19,513 (15764–23,587)	17.69 (14.29–21.38)	733 (388–988)	0.66 (0.35–0.9)	32,414 (17617–43,481)	29.38 (15.97–39.41)
	Female	1,441 (576–2,680)	2.66 (1.06–4.94)	7,101 (5695–8,611)	13.09 (10.5–15.87)	215 (43–318)	0.4 (0.08–0.59)	9,593 (2324–14,032)	17.68 (4.28–25.86)
	Male	2,409 (979–4,494)	4.3 (1.75–8.02)	12,412 (10068–15,031)	22.14 (17.96–26.81)	518 (263–765)	0.92 (0.47–1.36)	22,822 (12041–33,468)	40.71 (21.48–59.7)
50–54	Both	3,281 (1347–6,159)	2.71 (1.11–5.1)	21,901 (17646–26,392)	18.12 (14.6–21.84)	939 (467–1,287)	0.78 (0.39–1.06)	36,940 (19247–50,093)	30.56 (15.93–41.45)
	Female	1,206 (476–2,298)	2.02 (0.8–3.85)	7,993 (6387–9,687)	13.38 (10.69–16.22)	257 (46–388)	0.43 (0.08–0.65)	10,241 (2320–15,180)	17.15 (3.89–25.42)
	Male	2075 (879–3,954)	3.39 (1.44–6.47)	13,908 (11284–16,728)	22.75 (18.46–27.36)	682 (329–1,035)	1.12 (0.54–1.69)	26,698 (13220–40,161)	43.67 (21.62–65.69)
55–59	Both	2,680 (1114–5,260)	2.44 (1.01–4.78)	20,287 (16403–24,430)	18.45 (14.92–22.22)	981 (465–1,343)	0.89 (0.42–1.22)	33,953 (16755–46,274)	30.88 (15.24–42.09)
	Female	1,011 (413–2011)	1.84 (0.75–3.65)	7,509 (6011–9,102)	13.64 (10.92–16.53)	294 (53–446)	0.53 (0.1–0.81)	10,276 (2238–15,378)	18.66 (4.07–27.93)
	Male	1,669 (695–3,240)	3.04 (1.27–5.9)	12,778 (10397–15,362)	23.28 (18.94–27.99)	687 (342–1,012)	1.25 (0.62–1.84)	23,677 (12149–34,546)	43.14 (22.14–62.94)
60–64	Both	1989 (913–3,993)	2.72 (1.25–5.47)	14,179 (11532–16,999)	19.42 (15.8–23.29)	872 (427–1,190)	1.19 (0.58–1.63)	25,790 (12949–34,955)	35.33 (17.74–47.88)
	Female	762 (329–1,551)	2.09 (0.9–4.26)	5,235 (4231–6,289)	14.39 (11.63–17.29)	265 (43–393)	0.73 (0.12–1.08)	7,905 (1580–11,531)	21.73 (4.34–31.7)
	Male	1,227 (583–2,465)	3.35 (1.59–6.73)	8,944 (7339–10,667)	24.42 (20.04–29.13)	607 (284–886)	1.66 (0.78–2.42)	17,885 (8642–25,907)	48.83 (23.6–70.74)
65–69	Both	2,219 (1012–4,045)	2.89 (1.32–5.27)	14,881 (12131–17,799)	19.4 (15.81–23.21)	1,203 (537–1,636)	1.57 (0.7–2.13)	29,901 (13809–40,342)	38.98 (18–52.59)
	Female	892 (388–1,660)	2.29 (1–4.26)	5,614 (4538–6,722)	14.41 (11.65–17.25)	447 (79–641)	1.15 (0.2–1.65)	11,112 (2239–15,860)	28.52 (5.75–40.7)
	Male	1,327 (615–2,394)	3.52 (1.63–6.34)	9,267 (7608–10,986)	24.56 (20.16–29.11)	757 (340–1,119)	2 (0.9–2.96)	18,788 (8694–27,611)	49.78 (23.04–73.16)
70–74	Both	1941 (881–3,643)	3.64 (1.65–6.84)	10,602 (8658–12,522)	19.89 (16.25–23.5)	1,264 (577–1,674)	2.37 (1.08–3.14)	25,758 (12120–33,938)	48.33 (22.74–63.68)
	Female	811 (361–1,558)	2.95 (1.32–5.68)	4,068 (3307–4,875)	14.83 (12.05–17.77)	479 (111–693)	1.75 (0.4–2.53)	9,781 (2408–14,096)	35.65 (8.78–51.37)
	Male	1,130 (518–2,152)	4.37 (2.01–8.32)	6,534 (5366–7,699)	25.27 (20.75–29.78)	785 (378–1,153)	3.04 (1.46–4.46)	15,977 (7906–23,345)	61.79 (30.58–90.29)
75–79	Both	2068 (945–3,645)	6.24 (2.85–11.01)	7,010 (5773–8,276)	21.17 (17.43–24.99)	1,142 (523–1,502)	3.45 (1.58–4.53)	18,536 (8681–24,302)	55.97 (26.21–73.38)
	Female	913 (416–1,652)	5.21 (2.37–9.43)	2,779 (2263–3,311)	15.86 (12.92–18.9)	455 (83–644)	2.6 (0.47–3.68)	7,381 (1513–10,396)	42.14 (8.64–59.35)
	Male	1,155 (528–2034)	7.4 (3.38–13.04)	4,231 (3490–5,031)	27.12 (22.37–32.24)	687 (348–979)	4.4 (2.23–6.27)	11,155 (5757–15,815)	71.49 (36.9–101.36)
80–84	Both	3,404 (1842–5,441)	17.2 (9.31–27.49)	5,110 (4265–6,247)	25.82 (21.55–31.56)	978 (482–1,282)	4.94 (2.43–6.48)	12,407 (6253–16,172)	62.69 (31.6–81.71)
	Female	1,555 (821–2,469)	13.99 (7.38–22.21)	2,181 (1803–2,647)	19.62 (16.22–23.81)	425 (95–601)	3.82 (0.86–5.4)	5,389 (1311–7,571)	48.48 (11.79–68.11)
	Male	1848 (994–2,979)	21.31 (11.46–34.34)	2,928 (2441–3,591)	33.76 (28.13–41.4)	553 (271–763)	6.38 (3.12–8.8)	7,018 (3501–9,632)	80.9 (40.36–111.04)
85–89	Both	3,544 (2242–5,230)	37.21 (23.53–54.9)	3,624 (2925–4,560)	38.04 (30.7–47.87)	674 (325–865)	7.07 (3.42–9.08)	6,836 (3411–8,712)	71.76 (35.81–91.45)
	Female	1893 (1182–2,778)	31.31 (19.55–45.96)	1822 (1475–2,285)	30.14 (24.4–37.8)	274 (63–394)	4.53 (1.05–6.51)	2,794 (714–4,001)	46.22 (11.82–66.19)
	Male	1,651 (1047–2,481)	47.44 (30.08–71.27)	1802 (1446–2,277)	51.77 (41.53–65.42)	400 (209–556)	11.5 (6.01–15.96)	4,042 (2139–5,582)	116.11 (61.46–160.36)
90–94	Both	1814 (1239–2,604)	61.87 (42.25–88.8)	1756 (1375–2,255)	59.88 (46.91–76.91)	208 (103–268)	7.08 (3.51–9.15)	1871 (965–2,395)	63.8 (32.9–81.67)
	Female	1,193 (809–1,689)	56.2 (38.1–79.56)	1,070 (842–1,361)	50.38 (39.68–64.09)	103 (26–146)	4.87 (1.24–6.86)	939 (282–1,308)	44.21 (13.29–61.59)
	Male	621 (424–897)	76.76 (52.4–110.9)	686 (530–894)	84.83 (65.58–110.47)	104 (53–144)	12.89 (6.55–17.75)	932 (487–1,266)	115.21 (60.2–156.52)
95 plus	Both	535 (300–845)	83.68 (46.97–132.28)	639 (452–863)	99.91 (70.79–134.97)	38 (17–51)	5.9 (2.61–7.91)	335 (166–440)	52.45 (25.91–68.88)
	Female	412 (227–654)	79.52 (43.8–126.25)	465 (329–631)	89.74 (63.5–121.87)	25 (8–36)	4.84 (1.52–6.96)	224 (82–316)	43.18 (15.84–60.98)
	Male	123 (69–198)	101.52 (56.85–163.42)	174 (124–232)	143.48 (102.76–191.81)	13 (7–17)	10.46 (5.7–13.91)	112 (65–146)	92.19 (53.74–120.81)

**Figure 1:**
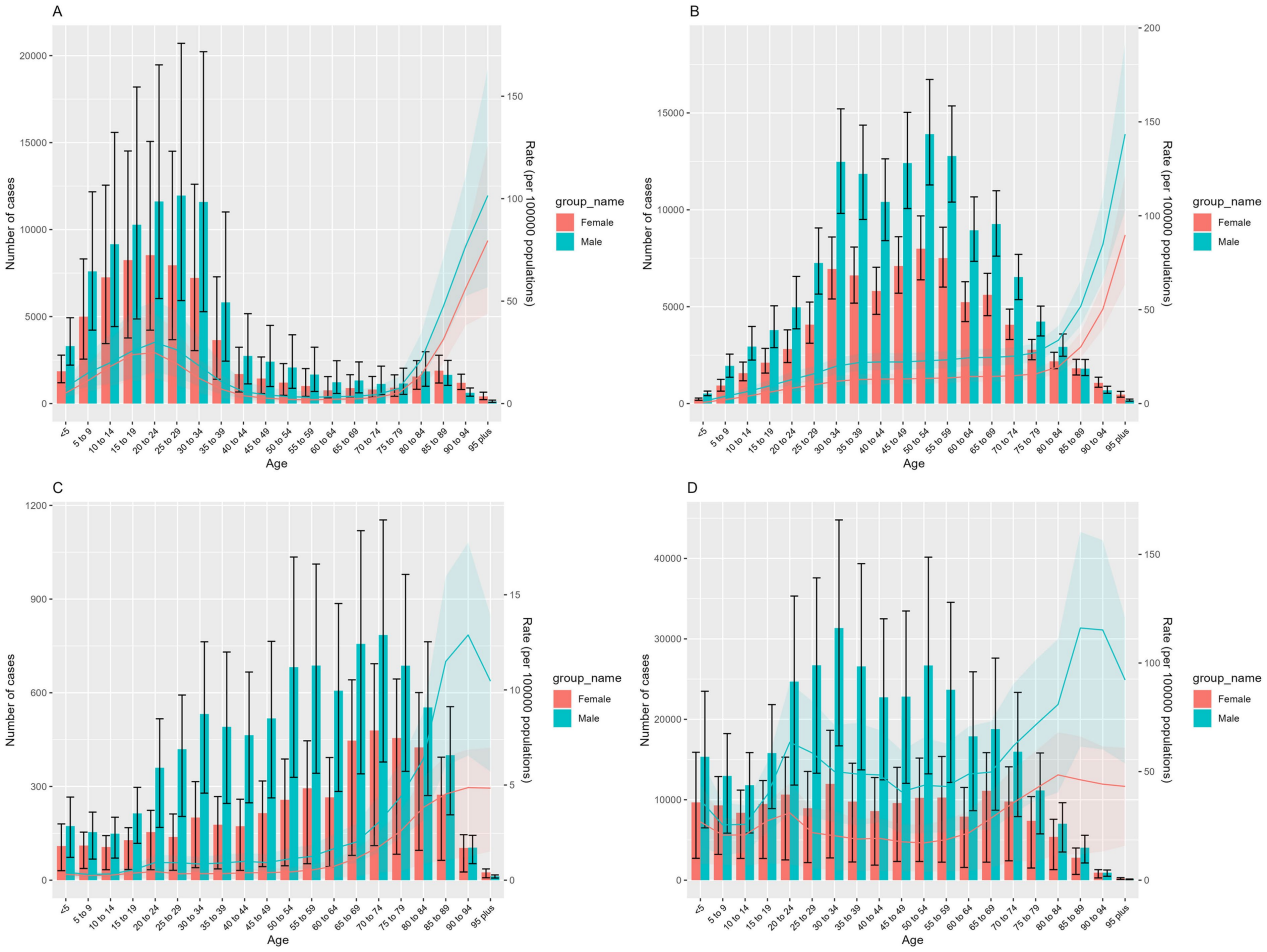
Disease burden of COP across different age groups in China in 2021: **(A)** number of incident cases and ASIR, **(B)** number of prevalent cases and ASPR, **(C)** number of deaths and ASMR, **(D)** number of DALYs and ASDR. Red represents females, and green represents males. Bars show the number of cases of incidence, prevalence, deaths, and DALYs. Error bars indicate the 95% uncertainty intervals for these numbers. Colored lines represent the age-standardized rates (ASRs) of incidence, prevalence, mortality, and DALYs. The shaded areas indicate the upper and lower bounds of the 95% uncertainty intervals for the ASRs.

New cases of COP were primarily observed in populations aged 5–34 years, while prevalence was concentrated among those aged 30–69 years. The majority of deaths occurred in the 50–79 years group, with the highest number of fatalities in the 70–74 years, accounting for 1,264 deaths and an ASMR of 2.37 (95% UI, 1.08–3.14) per 100,000. Additionally, the highest ASMR was seen in the 90–94 years age group, reaching 7.08 (95% UI, 3.51–9.15) per 100,000. The burden of DALYs due to COP was substantial in the 20–59 age group, with the highest DALYs observed in the 30–34 age group, totaling 43,329 (95% UI, 22,759–57,461), and an ASDR of 35.76 (95% UI, 18.79–47.43) per 100,000. Meanwhile, the highest ASDR was found in the 85–89 years subgroup at 71.76 (95% UI, 35.81–91.45) per 100,000 (Table 1; Figure 1).

### 3.2 Trends in the burden of COP in China from 1990 to 2021

**Figure 2:**
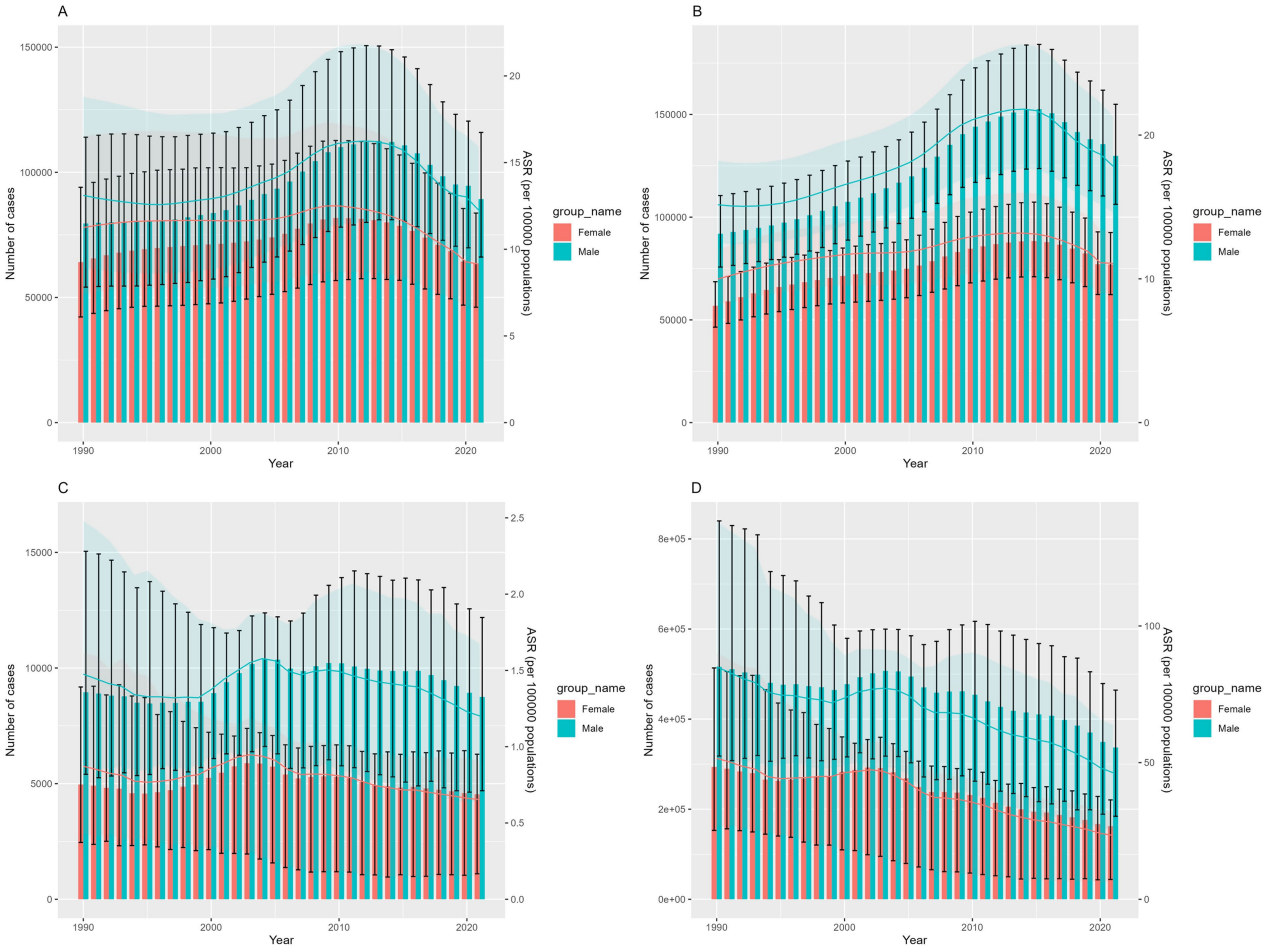
The trend of COP burden in China over time from 1990 to 2021: **(A)** number of incident cases and ASIR, **(B)** number of prevalent cases and ASPR, **(C)** number of deaths and ASMR, **(D)** number of DALYs and ASDR. Red represents females, and green represents males. Bars show the number of cases of incidence, prevalence, deaths, and DALYs. Error bars indicate the 95% uncertainty intervals for these numbers. Colored lines represent the age-standardized rates (ASRs) of incidence, prevalence, mortality, and DALYs. The shaded areas indicate the upper and lower bounds of the 95% uncertainty intervals for the ASRs.

From 1990 to 2021, the burden of COP for male patients in China was significantly higher than females, although the overall trends over time were consistent between the genders. Both the number of new cases and prevalent cases in men and women initially increased before subsequently declining, with the incidence peaking in 2012 and prevalence peaking in 2014 (Figures 2A,B). Additionally, the number of deaths in 2021 showed a fluctuating downward trend compared to 1990, with brief increases observed during 2000–2004 and 2006–2011 (Figure 2C). Similarly, DALYs exhibited a fluctuating downward trend from 1990 to 2021, though the decline was not significant between 2001 and 2010 (Figure 2D).

**Figure 3:**
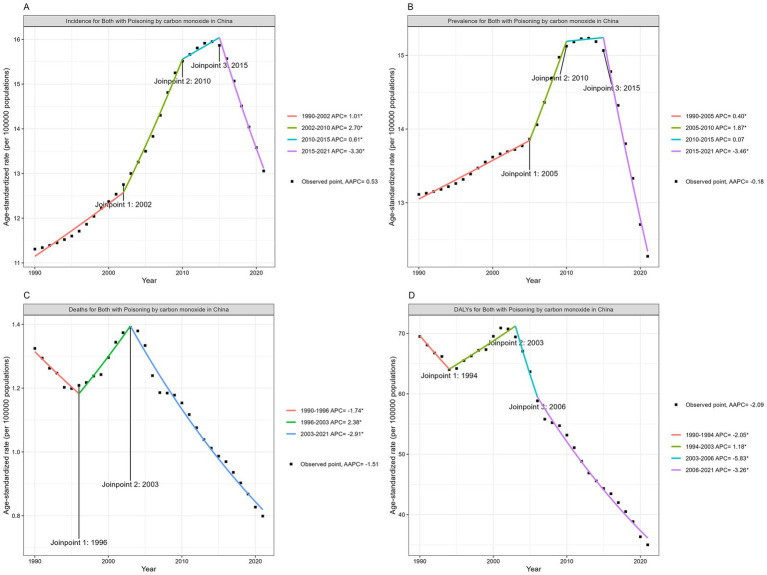
Joinpoint regression analysis: changing trend of ASIR **(A)**, ASPR **(B)**, ASMR **(C)** and ASDR **(D)** of COP in China from 1990 to 2021.

Joinpoint regression analysis revealed that from 1990 to 2021, the ASIR for COP in China exhibited an upward trend, with an AAPC of 0.53 (95% CI 0.48, 0.57). Segmental regression showed an increase in ASIR from 1990 to 2015 (1990–2002 APC = 1.01, 2002–2010 APC = 2.70, 2010–2015 APC = 0.61, *p* < 0.05), and a decline from 2015 to 2021 (2015–2021 APC = −3.30, *p* < 0.05) (Figure 3A). The ASPR showed a decreasing trend, with an AAPC of −0.18 (95% CI −0.21, −0.16). From 1990 to 2010, the ASPR increased (1990–2005 APC = 0.40, 2005–2010 APC = 1.87, *p* < 0.05), and decreased from 2015 to 2021 (2015–2021 APC = −3.46, *p* < 0.05) (Figure 3B). The ASMR demonstrated a downward trend, with an AAPC of −1.51 (95% CI -1.63, −1.39). From 1990 to 1996 and 2003 to 2021, the ASMR decreased, with an APC of −1.74 and −2.91, respectively, (*p* < 0.05). However, an upward trend was observed from 1996 to 2003, with an APC of 2.38 (*p* < 0.05) (Figure 3C). Between 1990 and 2021, the ASDR decreased, with an AAPC of −2.09 (95% CI -2.2, −1.96). Segmental regression indicated that the ASDR decreased during 1990–1994, 2003–2006, and 2006–2021, with respective APCs of −2.05, −5.83, and −3.26 (*p* < 0.05). An upward trend was observed from 1994 to 2003, with an APC of 1.18 (*p* < 0.05) (Figure 3D).

### 3.3 Age-period-cohort analysis of the burden of COP in China from 1990 to 2021

**Figure 4:**
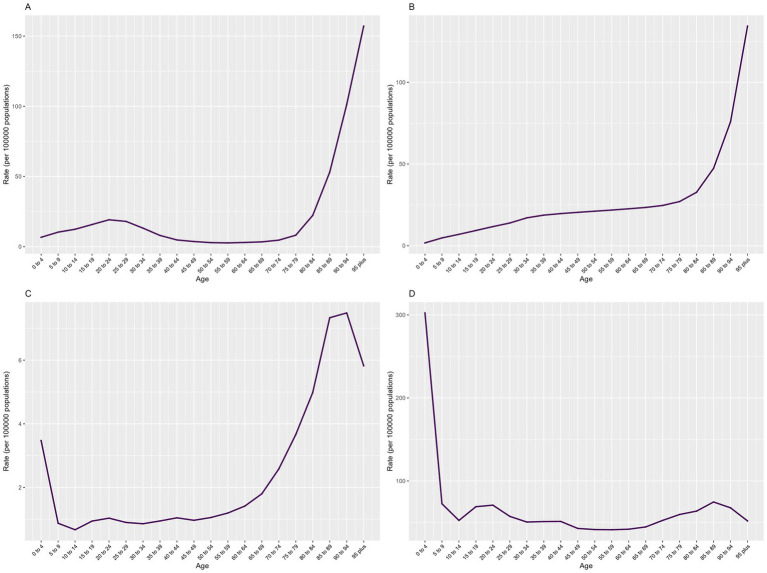
Age effects of COP burden in China from 1990 to 2021: **(A)** incidence, **(B)** prevalence, **(C)** mortality and **(D)** DALYs.

Effect of age: The incidence rate showed a slight upward trend in early life, peaking at 20–24 years of age, followed by a downward trend and a subsequent logarithmic increase starting at age 79. The prevalence rate generally increased with age, exhibiting exponential growth among individuals aged 79 and above. The mortality rate fluctuated considerably, with a high rate in the 0–4 age group, followed by a decline until age 14. Between ages 15 and 54, the mortality rate remained relatively stable with minor fluctuations, including small peaks in the 20–24 and 40–44 age groups. After age 55, the mortality rate began to increase exponentially, reaching a peak in 85–94 age group. The DALY rate was highest in the 0–4 age group, then declined significantly across subsequent age groups, with small peaks observed in the 20–24 and 85–89 age groups (Figure 4).

**Figure 5:**
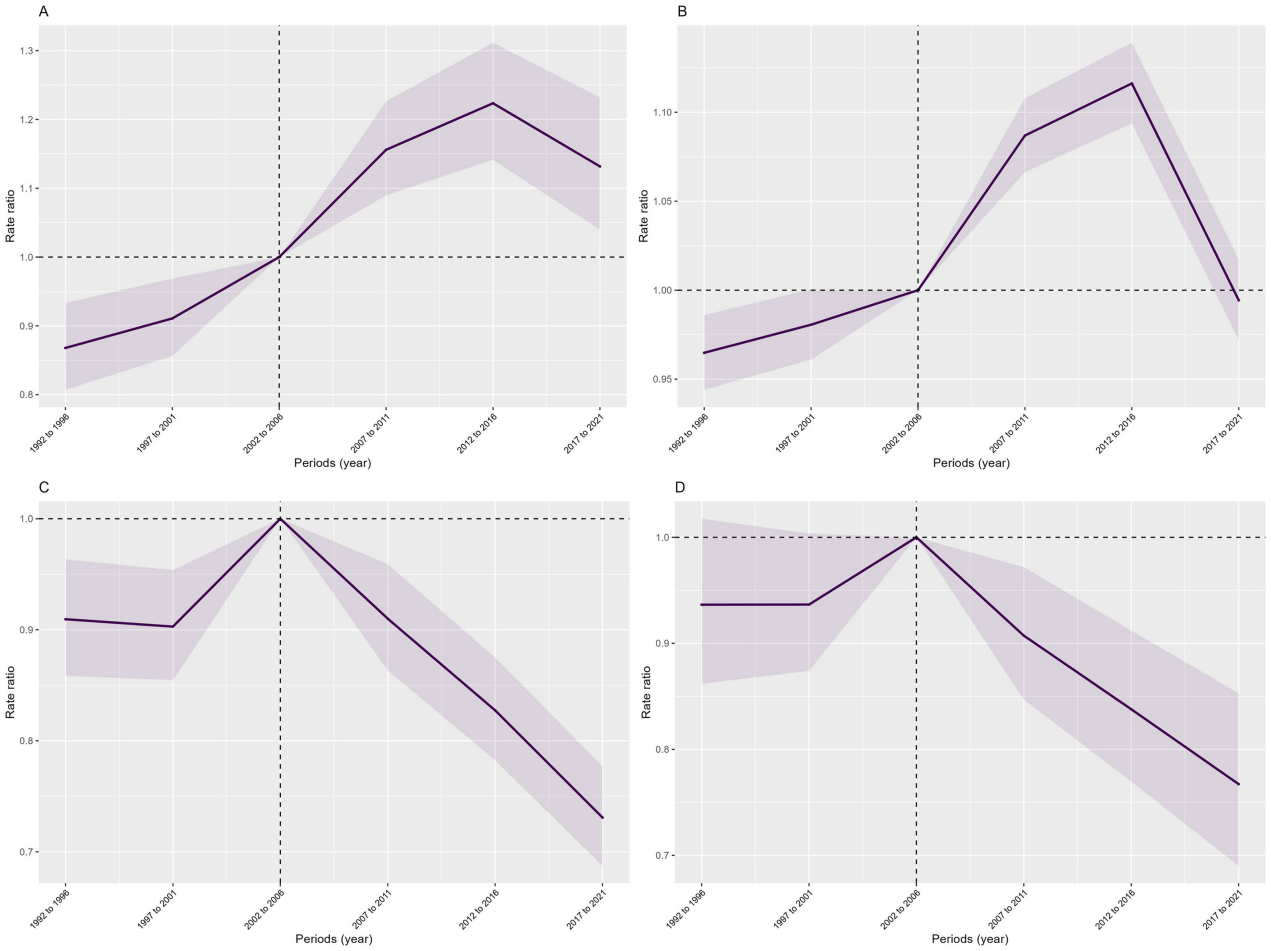
Period effects of COP burden in China from 1990 to 2021: **(A)** incidence, **(B)** prevalence, **(C)** mortality, **(D)** DALYs. The shaded areas indicate the upper and lower limits of the 95% confidence intervals of the rate ratio.

Effect of Period: The incidence and prevalence rates of COP fluctuated with changes over time. Using the year 2006 as the reference point (Relative Risk (RR) = 1.00), both the overall incidence and prevalence rates showed an upward trend from 1992 to 2016, peaking in 2016, followed by a declining trend from 2016 to 2021. Similarly, the mortality rate and DALY rate varied over time. With 2006 as the reference year (RR = 1.00), both the overall mortality rate and DALY rate exhibited an increasing trend from 2001 to 2006, reaching a peak in 2006, and then showing a decreasing trend from 2006 to 2021 (Figure 5).

**Figure 6:**
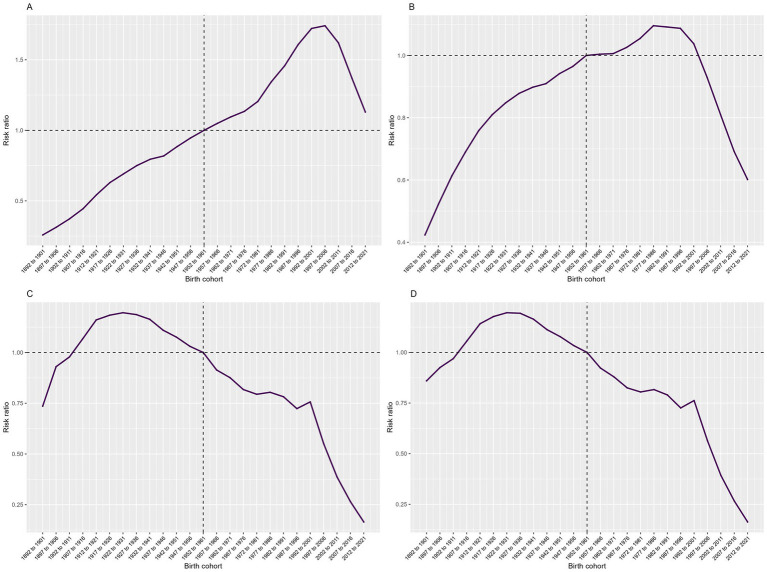
Cohort effects of COP burden in China from 1990 to 2021: **(A)** incidence, **(B)** prevalence, **(C)** mortality and **(D)** DALYs.

Effect of Cohort: Across the entire birth cohort, the cohort effect on the incidence rate of COP showed relatively minor fluctuations overall, with a significant upward trend from 1992 to 2006, peaking in 2006, and then declining to its lowest point during the period from 2006 to 2021. The prevalence rate exhibited more considerable fluctuations across cohorts, showing a significant and volatile upward trend from 1901 to 1986, peaking in 1986, and then declining to its lowest point from 1996 to 2021. The mortality rate and DALY rate generally showed a downward trend, with a significant increase from 1901 to 1931, reaching a peak in 1931, and then declining to the lowest point from 1931 to 2021 (Figure 6).

### 3.4 Forecasting the trends in the burden of COP in China from 2022 to 2050

**Figure 7:**
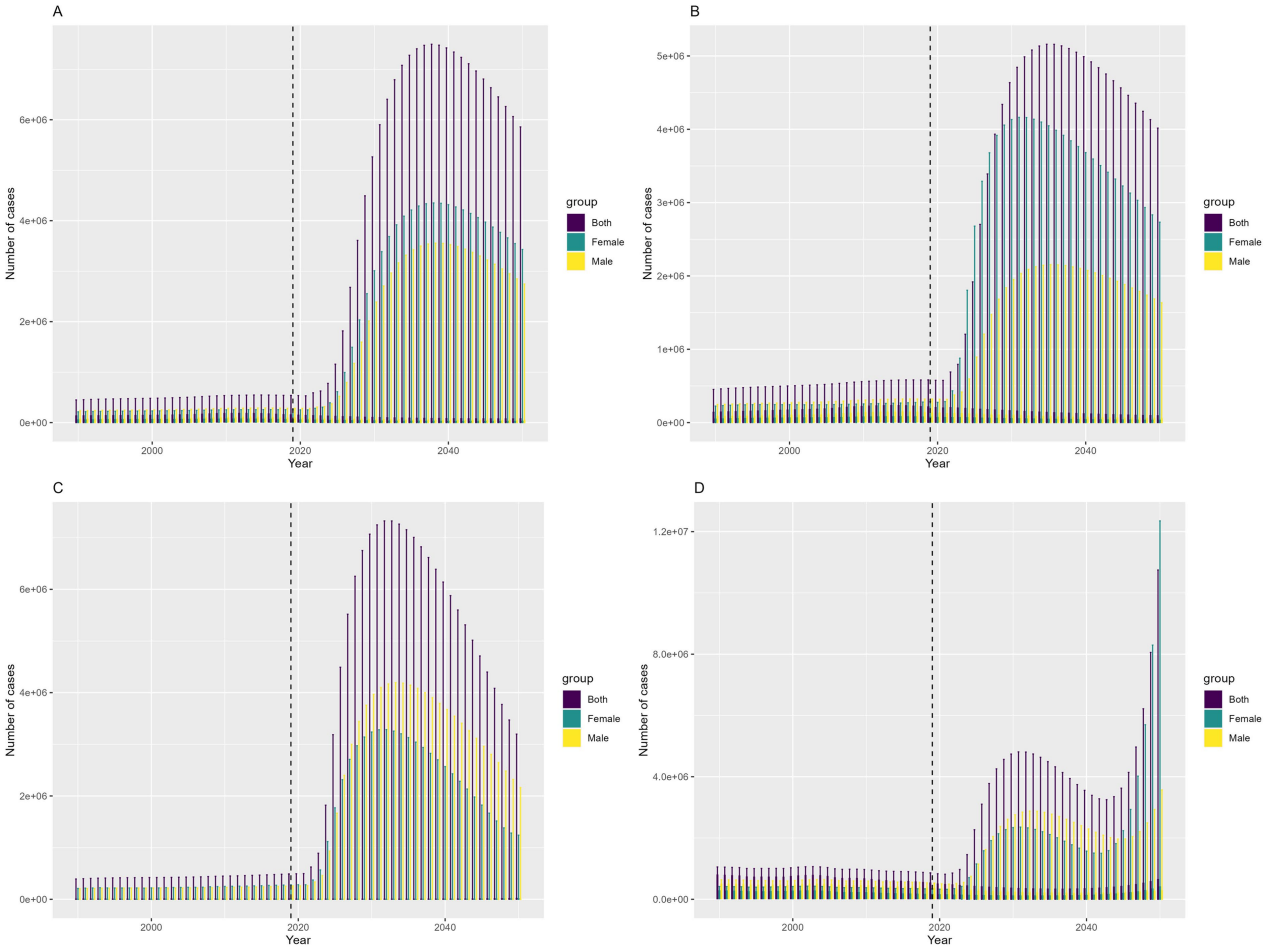
Prediction of the change trend of COP burden in China from 2022 to 2050: **(A)** number of incident cases, **(B)** number of prevalent cases, **(C)** number of deaths, **(D)** number of DALYs.

**Figure 8:**
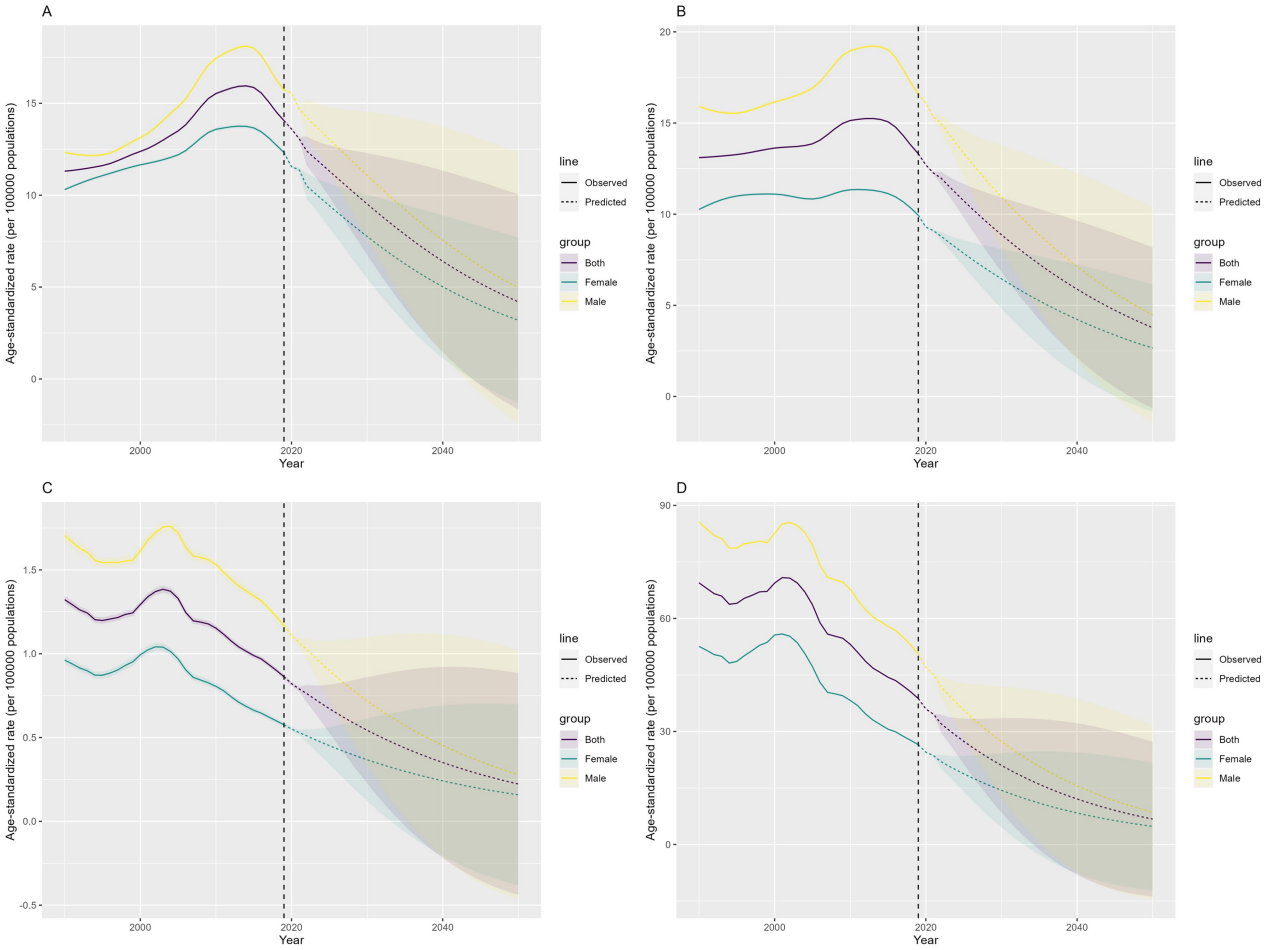
Prediction of the change trend of COP burden in China from 2022 to 2050: **(A)** ASIR, **(B)** ASPR, **(C)** ASMR, **(D)** ASDR. Yellow represents males, green represents females, and purple represents both sexes. The shaded areas indicate the upper and lower limits of the 95% uncertainty intervals.

According to predictions from the Bayesian Age-Period-Cohort model, the number of incidence, prevalence, deaths, and DALYs related to COP in China are expected to increase from 2022 to 2050, peaking in 2038. After 2038, a yearly decline is projected, with similar trends observed in both males and females (Figure 7). However, the ASRs are projected to decline. The ASIR is predicted to decrease from 12.40 per 100,000(95% UI, 11.61–13.20) in 2022 to 4.20 (95% UI, −4.20–10.05) in 2050. The ASPR is expected to decline from 11.98 per 100,000 (95% UI, 11.63–12.34) in 2022 to 3.77 (95% UI, −1.64–8.18) in 2050. The ASMR is forecast to decrease from 0.76 per 100,000 (95% UI, 0.71–0.81) in 2022 to 0.22 (95% UI, −0.44–0.88) in 2050. Finally, the ASDR is expected to drop from 32.09 per 100,000 (95% UI, 29.72–34.45) in 2022 to 6.75 (95% UI, −13.80–27.30) in 2050 (Figure 8).

## 4 Discussion

This study presents a comprehensive analysis of the disease burden associated with COP in China over three decades and projects future trends through 2050. The findings reveal dynamic temporal patterns in COP burden, characterized by an initial rise in incidence and prevalence followed by a decline in recent years, alongside a general downward trajectory in mortality and DALYs. Notably, in 2021, the highest DALYs were observed among individuals aged 30–34 years, while older populations exhibited heightened vulnerability to severe outcomes. Despite overall improvements in ASRs over the study period, projections suggest a potential resurgence in COP-related burden in the coming decades, peaking around 2038.

The evolving burden of COP in China reflects a complex interplay of socioeconomic progress, demographic shifts, and changing risk factors. Overall, despite fluctuations, the rates have been decreasing in recent years, aligning with global trends. Global burden of disease analysis showed that over the past 21 years, the global number of unintentional COP deaths have significantly decreased (8, 15). The observed temporal trends, characterized by initial increases in incidence and prevalence followed by declines, and decreases in mortality and DALYs, may be linked to China’s energy transition and the implementation of public health interventions (15, 16). The shift from coal-dependent to cleaner energy systems likely reduced household and occupational exposure to CO, contributing to the downward trajectory of severe outcomes (17, 18). Furthermore, public health measures such as stricter supervision of appliances that produce CO gas and health education can further mitigate the risk of COP. In addition, the notable reductions in mortality risk and DALYs may be attributed to the ongoing optimization and advancement of treatment strategies, particularly the widespread use of hyperbaric oxygen therapy (HBOT). Prior studies have demonstrated the beneficial effects of HBOT on COP (19–23). A multicenter retrospective study involving over 20,000 COP patients indicated that HBOT could improve short- and long-term survival (24), and a prospective randomized trial demonstrated that it could also enhance neurological outcomes (25). In recent years, novel treatments such as phototherapy and extracorporeal membrane oxygenation have also been introduced (1). With continuous refinement of treatment approaches, it is believed that the burden of COP will continue to decline.

Although the overall burden of COP in China has decreased in recent years, the death risk remains higher than the global average (0.366 per 100,000 population) (8). It is also higher than America. In the US, total CO-related deaths decreased from 1,253 in 2015 to 1,067 in 2021, with crude death rates declining from 0.39 to 0.35 per 100,000 (26). Mortality rates could be influenced by various factors, including levels of national development, public health management, treatment strategies and environmental and climate conditions. Although China has experienced rapid development as a developing country, there are still many areas that require optimization when compared to developed nations. For example, multiple states and regions in the US have enacted legislation mandating CO alarms in residences, which has helped raise public awareness of COP risks and promote the widespread adoption of alarms (26). However, China has yet to implement similar policies. This highlights significant room for improvement in this area within China.

In terms of gender, we observed a higher burden of COP among males compared to females, which aligns with previous studies (8, 15, 27–29). A key explanation for this phenomenon may lie in exposure disparities between genders. Previous research indicates that occupations with higher risks of COP, including those requiring proximity to combustion sources (e.g., engines and fires) or CO-emitting equipment (e.g., firefighting apparatus, diesel and forklift engines, and mechanical machinery), have a greater proportion of male employees (28, 29). In addition, drinking might increase the risk of COP. People affected by alcohol are also less likely to recognize the early symptoms of COP (30, 31). Additionally, gender differences may influence both the severity of poisoning and post-COP prognosis, with females demonstrating superior outcomes compared to their male counterparts, particularly among premenopausal couples (32). The specific pathophysiological mechanism of this phenomenon is still unclear and requires further investigation.

This study showed that in 2021, the 30–34 age group experienced a substantial burden of COP, suffering the greatest loss of life years, and this finding aligns with global burden of disease analysis results (8, 33). Populations aged 30–34 are often the primary breadwinners in their families, and their exposure risks are relatively high both in work environments and at home. Additionally, according to the DALY age-weighting calculation rules, premature death and disability in this age group are assigned high social value weights. Nevertheless, population in this age group did not face the highest risk of death. Mortality rates were higher among older populations aligns with other studies (26), with the greatest number of deaths occurring in the 70–74 age group and the highest ASMR observed in the 90–94 age group. This may be related to the presence of multiple underlying diseases, depletion of physiological reserves, and atypical clinical presentations that are prone to misdiagnosis for older people. Therefore, greater attention is needed for the prevention and treatment of COP in the older population.

According to predictions from the Bayesian Age-Period-Cohort model, the number of incident cases, prevalence, deaths, and DALYs related to COP in China is expected to increase from 2022 to 2050, peaking around 2038. However, the ASRs are projected to decline. This discrepancy may be attributed to the fact that numbers and ASRs reflect different information and are influenced by distinct factors. Given that the older population faces a high risk of COP, the increase in numbers may primarily due to factors such as population growth and population aging in China (34, 35). Nevertheless, the decline in ASRs indicates that, after accounting for changes in the population’s age structure, the burden of COP on individual health is expected to decrease, suggesting that prevention and control measures targeting COP might have been effective. However, the possible increase in numbers highlights that the overall demand for resources and services to combat this disease may continue to grow, with the older population remaining the primary focus for COP prevention, monitoring, and treatment.

This study analyzes the changing patterns of the disease burden caused by COP in China from 1990 to 2021 and forecasts the epidemiological trends from 2022 to 2050. The findings provide data support for the prevention and control of COP in China and other developing countries. However, certain limitations should be noted: Firstly, the data sources and acquisition methods of the GBD database are not entirely consistent, which may lead to some biases in the results. Secondly, the GBD database only publishes data at the national level, and therefore it was not possible to further analyze the disease burden of COP in different provinces or regions in China.

## 5 Conclusion

From 1990 to 2021, China has experienced dynamic temporal patterns in the burden of COP, characterized by an initial rise in incidence and prevalence, followed by a decline in recent years, alongside a general downward trajectory in mortality and DALYs. Additionally, projections indicate a potential resurgence in COP-related burden in the coming decades.

## Data Availability

The raw data supporting the conclusions of this article will be made available by the authors, without undue reservation.
