# Correction: The burden of carbon monoxide poisoning in China from 1990 to 2021 and forecasts to 2050

**DOI:** 10.3389/fpubh.2025.1699926

**Published:** 2025-09-15

**Authors:** Frontiers Production Office

**Affiliations:** Frontiers Media SA, Lausanne, Switzerland

**Keywords:** carbon monoxide poisoning, the Global Burden of Diseases, Injuries, and Risk Factors Study (GBD), incidence, prevalence, mortality, Disability Adjusted Life Years

For reviewer Shaden Salameh the affiliation “Hadassah University Hospital Mount Scopus, Israel” was erroneously given as “Institute of Gastroenterology and Hepatology, Hadassah Medical Center, Israel”

The original version of this article has been updated.

